# Trimethoprim-sulfamethoxazole induced eosinophilic pneumonia: A case report

**DOI:** 10.1016/j.rmcr.2022.101632

**Published:** 2022-03-15

**Authors:** Yasutaka Mochizuka, Tomoyuki Fujisawa, Yusuke Inoue, Hironao Hozumi, Yuzo Suzuki, Masato Karayama, Kazuki Furuhashi, Noriyuki Enomoto, Yutaro Nakamura, Naoki Inui, Takafumi Suda

**Affiliations:** a Second Division, Department of Internal Medicine, Hamamatsu University School of Medicine, Hamamatsu, Japan; b Department of Clinical Pharmacology and Therapeutics, Hamamatsu University School of Medicine, Hamamatsu, Japan

**Keywords:** Eosinophilic pneumonia, Organizing pneumonia, Trimethoprim-sulfamethoxazole, Adverse reaction

## Abstract

We report herein a case of trimethoprim-sulfamethoxazole (TMP-SMX) induced eosinophilic pneumonia in a 27-year-old woman with radiological features of bilateral nonsegmental airspace consolidation resembling cryptogenic organizing pneumonia at the peripheral lung fields. Organizing pneumonia with eosinophil infiltration in the lung specimens and marked eosinophilia in the peripheral blood and bronchoalveolar lavage fluid were observed. Discontinuation of TMP-SMX improved eosinophilia and radiological abnormality, which confirmed the association between the use of TMP-SMX and onset of eosinophilic pneumonia. Although TMP-SMX induced eosinophilic pneumonia is not common, clinician should be aware that drug-induced eosinophilic pneumonia could happen during the course of TMP-SMX administration.

## 1 Introduction

Trimethoprim-sulfamethoxazole (TMP-SMX) is a synthetic sulfonamide antimicrobial agent mainly used for the treatment and/or prevention of *Pneumocystis* pneumonia and uncomplicated cystitis. Sulfonamides have a relatively high incidence of adverse effects including gastrointestinal and blood disorders, rash, and hypersensitivity. However, reports of drug-induced pneumonia are rare [1]. There are a few reports of TMP-SMX induced pneumonia [2,3] causing bilateral ground glass opacity on chest computed tomography (CT) in majority of the cases.

Eosinophilic pneumonia is characterized by a marked accumulation of eosinophils in the lung [4]. Although the exact etiology is unknown, secondary drug-induced eosinophilic pneumonia is implicated with many medications [5]. Here we describe a case of TMP-SMX-induced eosinophilic pneumonia with radiological features of bilateral nonsegmental airspace consolidation in the peripheral lung fields resembling cryptogenic organizing pneumonia.

## 2 Case presentation

**Fig. 1:**
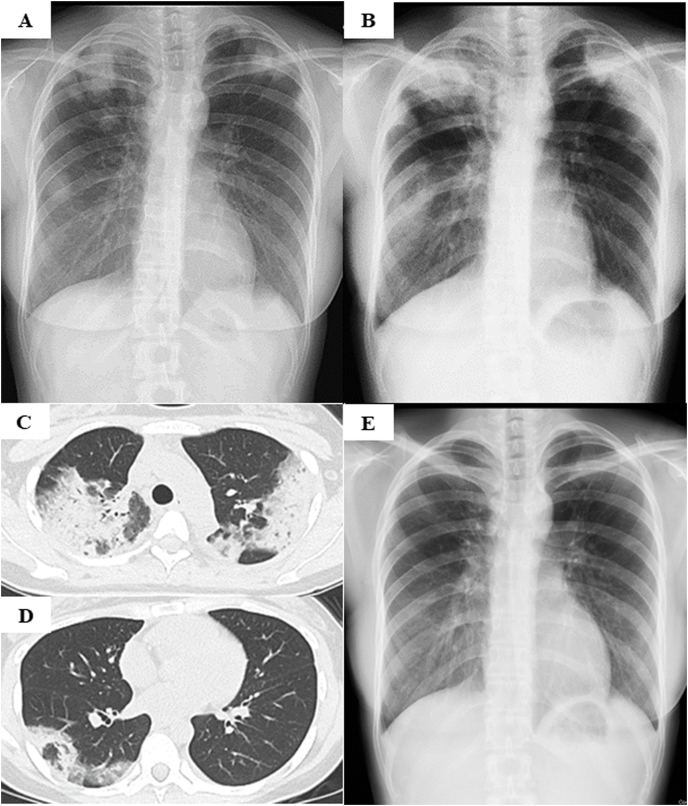
Chest radiograph showing the bilateral infiltrative shadows in upper lung fields (A). After 1 week of levofloxacin administration, the infiltrative shadows increased (B), and chest computed tomography show nonsegmental bilateral airspace consolidation in the peripheral region (C, D). After one month of TMP-SMX discontinuation, abnormal infiltrates almost disappeared (E). TMP-SMX; trimethoprim-sulfamethoxazole.

A 27-year-old Japanese woman admitted to our hospital complained of cough and dyspnea. She had no smoking history. She had a history of childhood asthma and brain tumor surgery. A brain biopsy was performed two months ago to exclude the recurrence of her brain tumor. After the biopsy, she developed a wound infection and was treated with intravenous meropenem 3000 mg/day and linezolid 1200 mg/day for one month, followed by oral TMP-SMX (320 mg–1600 mg/day). After 3 weeks of TMP-SMX treatment, she presented with cough and shortness of breath without fever. There was no evidence of skin rash, cervical or supraclavicular lymphadenopathies, and arthralgia. Laboratory data showed elevated white blood cell count (13,130/μL) with marked eosinophilia (2717/μL) in peripheral blood and elevated serum C-reactive protein level (7.09 mg/dL). The result of the autoimmune screening was negative. Chest radiographs showed bilateral infiltrates mainly in upper lung fields (Fig. 1A). Despite administration of levofloxacin 500 mg/day for a week, her symptoms worsened and infiltrative shadows increased on chest radiographs (Fig. 1B). Chest CT showed bilateral nonsegmental airspace consolidation in the peripheral region of the lung (Fig. 1C and D). Pulmonary function tests showed decreased forced vital capacity (FVC), 1.80 L (59%).

**Fig. 2:**
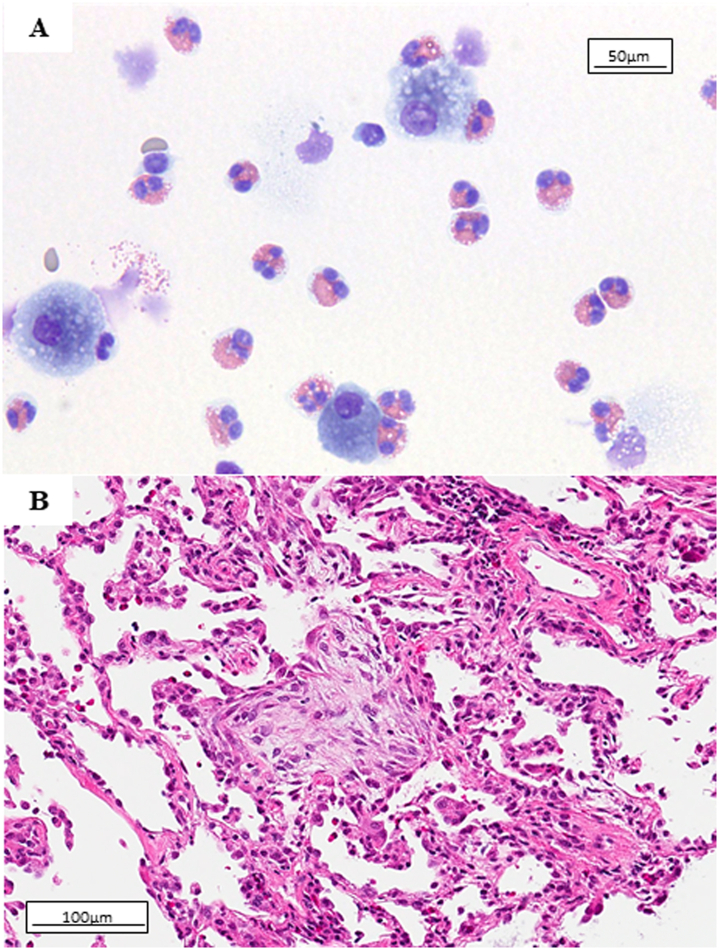
Bronchoalveolar lavage cytology showing eosinophils (A, hematoxylin eosin stain). Histological examination of transbronchial lung biopsy from the right lower lobe showing organizing pneumonia with mild infiltration of eosinophils (B, hematoxylin eosin stain).

Bronchoscopic examination showed increased eosinophils in bronchoalveolar lavage fluid (BALF) (Fig. 2A) (70.6% of eosinophils in the differential cell count). Histological examination on transbronchial lung biopsy from the right lower lobe revealed organizing pneumonia with mild infiltration of eosinophils (Fig. 2B). Workup for infectious etiology was negative. After discontinuation of TMP-SMX, her general condition improved day-by-day and peripheral blood eosinophils were decreased without additional treatments. According to the clinicopathological findings, we diagnosed the patient with TMP-SMX induced eosinophilic pneumonia.

After one month of discontinuation of TMP-SMX, the infiltrative shadows on the chest radiograph almost disappeared (Fig. 1E), and FVC increased to 2.45 L (77.8%). Since then, she had no relapse of pneumonia.

## 3 Discussion

In this report, we described a case of TMP-SMX induced eosinophilic pneumonia. After 3-week of oral administration of TMP-SMX, the patient had eosinophilia in peripheral blood and BALF, and radiological and pathological findings consistent with eosinophilic organizing pneumonia. The improvement after discontinuation of the drug indicated that the patient had drug-induced pneumonia caused by TMP-SMX. Based on the clinicopathological findings, the patient was diagnosed with TMP-SMX-induced eosinophilic pneumonia.

The major causes of secondary eosinophilic pneumonia include drugs and toxins [5]. Sulfonamides are common allergies, which sometimes causes increased eosinophils in peripheral blood and lung. Parry et al. reviewed the 50 cases of sulphasalazine-induced lung diseases [6]. Among them, 26 (52%) of the cases showed peripheral eosinophilia and five of 11 cases who underwent BAL revealed eosinophilia in BALF. In our case, marked eosinophilia in peripheral blood and BALF were observed, and was subsequently resolved by discontinuation of TMP-SMX, suggesting that sulfamethoxazole is the trigger for eosinophilic pneumonia.

This case demonstrated characteristic radiological features of bilateral nonsegmental airspace consolidation in the chest CT, which are reminiscent of cryptogenic organizing pneumonia or chronic eosinophilic pneumonia. Additionally, lung pathological findings also showed organizing pneumonia with eosinophilic infiltration. In terms of radiologic characteristics of TMP-SMX-induced pneumonia, major findings are ground glass opacity and reticular opacity, but airspace consolidation predominantly in the peripheral lung region is rare [2,3,[7], [8], [9]]. The case series of 10 adults of interstitial lung disease due to TMP-SMX demonstrated that major radiographic abnormalities were ground glass opacity and patchy area of infiltrations [9]. Although radiological findings in this case, bilateral nonsegmental airspace consolidation in the peripheral lung fields, were not common as TMP-SMX-induced pneumonia, a history of TMP-SMX exposure and improvement with discontinuation confirmed the diagnosis of drug-induced pneumonia by TMP-SMX.

## 4 Conclusion

In conclusion, we describe a case of eosinophilic pneumonia caused by TMP-SMX with radiological features of bilateral nonsegmental airspace consolidation. Marked eosinophilia and bilateral airspace consolidation were improved after discontinuation of TMP-SMX, supporting the association between the use of TMP-SMX and the onset of eosinophilic pneumonia. Although TMP-SMX induced eosinophilic pneumonia is not common, clinician should be aware drug-induced eosinophilic pneumonia could happen during TMP-SMX administration.

## Consent to publish statement

Written informed consent was obtained from the patient for publication on this case report and any accompanying images.

## Financial support

None.

## Author contoributions

TM, TF: conception and design of the study, acquisition of data, analysis and interpretation of data, manuscript writing. YI, HH, YS, MK, KF, NE, YN, NI, TS: analysis and interpretation of data, and revising the manuscript. All authors reviewed and approved the manuscript.

## Declaration of competing interest

The authors have no conflicts of interest to declare.
